# Vitamin D status and associations with diet, objectively measured physical activity patterns and background characteristics among adolescents in a representative national cross-sectional survey

**DOI:** 10.1017/S1368980022000222

**Published:** 2022-06

**Authors:** Eva Warensjö Lemming, Jessica Petrelius Sipinen, Gisela Nyberg, Lotta Moraeus, Anna Karin Lindroos

**Affiliations:** 1 Department of Risk and Benefit Assessment, Swedish Food Agency, Uppsala 75126, Sweden; 2 The Swedish School of Sport and Health Sciences (GIH), Stockholm, Sweden; 3 Karolinska Institutet, Department of Global Public Health, Stockholm, Sweden; 4 Department of Internal Medicine and Clinical Nutrition, The Sahlgrenska Academy, University of Gothenburg, Gothenburg, Sweden

**Keywords:** Riksmaten, Adolescents, 25-hydroxyvitamin D, Diet, Vitamin D, National survey

## Abstract

**Objective:**

To report on vitamin D status, measured as plasma 25-hydroxyvitamin D concentration (25(OH)D), the prevalence of vitamin D insufficiency and deficiency, and to explore associations between vitamin D status and background characteristics.

**Design:**

Data were collected in a National Dietary Survey, Riksmaten adolescents 2016–2017. The participants completed dietary assessments and questionnaires on the web and wore accelerometers. (25(OH)D) was measured with a MS method.

**Setting:**

Representative survey conducted in schools throughout Sweden.

**Participants:**

Participants attended school years 5 (Y5, mean age 12. 5 years), 8 (Y8, mean age 14. 5 years) and 11 (Y11, mean age 18 years), and included 1100 participants.

**Results:**

Overall, there was no difference in plasma 25(OH)D between girls and boys. Vitamin D insufficiency differed between the three school years. The prevalence of insufficiency in Y5 was 32 (boys) and 48 (girls) percent, while in Y11 62 (boys) and 43 (girls) percent. The prevalence of deficiency in Y11 was 16 and 15 % in boys and girls, respectively. Being born outside of Sweden was associated with a 10-fold increased risk of being vitamin D deficient. Deficiency was also associated with longer time spent in sedentary intensity, a lower consumption of fortified dairy products and fats and oils.

**Conclusions:**

Vitamin D deficiency was most common in the oldest age group and being born outside of Sweden increased the risk of being deficient. The present study will form a baseline for future follow-up studies of the implementation of a new mandatory vitamin D fortification policy in 2018.

Vitamin D is needed for the metabolism of Ca and P and is important for the mineralisation of the skeleton and teeth. The hallmark of vitamin D deficiency is rickets in children and osteomalacia in adults. There is evidence that vitamin D may exert effects on extraskeletal organs and tissues. Associations with cancer, CVD, high blood pressure, type 2 diabetes, immune response, infectious disease, neuropsychological function and others have been reported^(1–4)^. However, the evidence for a causal link to some of these outcomes is not entirely clear, and large randomised controlled trials have largely failed to confirm the beneficial effects of vitamin D supplementation on different outcomes^(5)^. Mendelian randomisation studies have also failed to confirm causal links^(6,7)^.

Vitamin D can be obtained by cutaneous synthesis from exposure to sunlight, from dietary sources or from supplements. With optimal sunlight exposure, there is no need of another source of vitamin D. However, in countries located on northern latitudes, like Sweden, the cutaneous synthesis of vitamin D is diminished during the winter months (November through April) and food sources become more important to maintain status. In Sweden, there is mandatory fortification of dairy products (all types of milk, yogurt and sour milk) and of margarine and fat blends. The current policy on vitamin D fortification was implemented in 2018 because population intakes were low^(8)^. However, prior to 2018, milk as well as margarine and fat blends were fortified with vitamin D, but in a lower concentration^(9)^. The recommended intake for vitamin D is 10 µg per day for all age groups between 2 and 75 years of age according to the Nordic Nutrition Recommendations^(1)^, and the estimated average requirements is set to 7·5 µg per day. The reasoning behind the recommended intake is that in order to maintain a sufficient serum 25(OH)D concentration (>50 nmol/l) during the winter months in a majority of the population, a vitamin D intake of 10 µg is needed^(1)^. In the national representative survey, Riksmaten adolescents 2016–2017, the proportion of participants with a vitamin D intake below the average requirement was above 80 % among girls in all three age groups of the survey (school years 5 (mean age 12·5 years), 8 (mean age 14·5 years) and 11 (mean age 18 years)), and among the youngest boys. Among boys in school year 8 and 11, the proportion with an intake below the average requirement was 60 and 71 %, respectively^(10)^. Thus, the prevalence of inadequate vitamin D intake was very high among these adolescents, which could in theory translate into low vitamin D status and risk of vitamin D deficiency, measured as total plasma 25-hydroxyvitamin D concentration (25(OH)D) < 30 nmol/l (Nordic Nutrition Recommendations^(10)^). However, a number of other non-dietary factors, such as sun exposure, living conditions, sunbathing behaviour and age influence vitamin D status^(3)^, and 25(OH)D concentration, especially winter concentration, is largely genetically determined^(11)^. It is estimated that around 10 % of the variance of the 25(OH)D concentration is related to diet^(3)^. In a previous smaller study including 200 school children, aged 10–12 years, living in major cities throughout Sweden, 5 % had 25(OH)D concentration <30 nmol/l and 42 % had 25(OH)D concentration <50 nmol/l^(8)^. In this study, there was no difference in status between boys and girls, and lower 25(OH)D concentrations were associated with higher BMI and being born outside Europe^(8)^. The results from a previous UK-based study indicated that adequate sunlight exposure prevents winter vitamin D deficiency in 83 % of healthy adolescents of white origin, while this was not true among working adults of south Asian origin^(12)^. Against this background, the aim of the present study is therefore to report on the plasma 25(OH)D status in a representative national cross-sectional sample, Riksmaten adolescents 2016–2017, and to investigate the prevalence of vitamin D insufficiency and deficiency. The study will also explore associations between plasma 25(OH)D status and diet, objectively measured physical activity and other background characteristics.

## Study design and population

Riksmaten adolescents 2016–2017 is a nationally representative cross-sectional dietary survey conducted by the Swedish Food Agency and carried out between September 2016 and May 2017^(13)^. Participants were recruited from classes at representative schools throughout Sweden and attended the school years 5 (mean age 12·5 years), 8 (mean age 14·5 years) and 11 (mean age 18 years). The participants completed dietary assessment, and questionnaires regarding background, lifestyle and food propensity on the web. Study personnel measured participants’ weight and height, and participants wore an accelerometer on the hip for seven consecutive days for the assessment of physical activity and sedentary time. Blood samples were collected in a subsample. The collection of data, except for the completion of the dietary assessment and questionnaires, were done during the school visit of the survey. Altogether, 5145 pupils at randomly selected schools were invited to participate. In total, 3477 individuals (68 %) participated in some stage of the survey, and 3099 participants (60 %) provided complete information on diet. In the subsample, the overall response rate was 55 %, and 1105 (46 %) participants had provided blood and complete information on diet. Valid vitamin D measurement (described below) was available for 1100 participants and is the study sample of the present study.

## Blood samples

The blood samples were collected in non-fasting participants in collaboration with the regional divisions for Occupational and Environmental Medicine in Sweden throughout the entire time of the survey (September through May). The blood samples were immediately centrifuged and placed in a freezer. All samples were subsequently stored at –80°C until analysis.

## Vitamin D analysis

Total plasma 25(OH)D concentration, including 25(OH)D_3_ and 25(OH)D_2_, was determined with HPLC atmospheric pressure chemical ionisation (APCI) MS at Vitas, Oslo, Norway (www.vitas.no) as previously described^(14)^. The CV for interassay analyses was around 7 %. The method is accredited by the Vitamin D External Quality Assessment Scheme. The sum of 25(OH)D_3_ and 25(OH)D_2_ equals the total plasma 25(OH)D concentrations among the participants. A three-level categorical variable called vitamin D status, based on total plasma 25(OH)D concentrations, were generated and coded as 0 < 30 nmol/l, 1 between 30 and 50 nmol/l and 2 > 50 nmol/l.

## Dietary assessment

Participants recorded their consumption of foods and beverages in a web-based method called RiksmatenFlexDiet^(13)^, which has been shown valid for use in an adolescent population^(15)^. The consumption was recorded during 2 d (data collection days 1 and 3), which were non-consecutive and retrospective. RiksmatenFlexDiet builds on the 24-h recall technique and requires the participants to record all food and beverages consumed in the preceding 24 h^(15)^. The second day of data collection was the day of the school visit and the day when participants provided blood samples and consecutive to day 1. Day 3 was a random day occurring 3–9 d after day 2. Complete diet information means that valid diet data were available from at least days 1 and 3, but most participants had information from all 3 d (96 %). RiksmatenFlexDiet contains all information needed for the registration, including a picture portion guide, a search tool to search for the foods and beverages (food list) consumed and automatic prompts to remember items easily forgotten. Participants were asked to add information on time, place and meal type to each meal. The food list contained 778 typical foods and beverages, linked to the Swedish national food composition database (version Riksmaten adolescents 2016–2017) for the calculation of energy intake and nutrient intakes per day. The content of vitamin D in the database includes both vitamin D_2_ and D_3_. Data from the two retrospective days were used in the current study in order to avoid mixing retrospective and prospective methods.

## Energy intake and misreporting

To evaluate misreporting status in the study, estimated energy intake was compared with the total energy expenditure according to the methods of Goldberg and Black^(16,17)^. Total energy expenditure was calculated using equations for BMR^(18)^, information on physical activity from accelerometers^(19)^ and assuming a diet-induced thermogenesis of 10 %. As previously described^(10)^, each participant was classified into under-, plausible- or over-reporter of energy and used as a categorical variable in the analysis.

## Other variables

Information on different background characteristics was collected in the web-based questionnaire, RiksmatenFlexQuestionnaire (FlexQ). Highest educational degree of either parent was used and five levels of education were classified into ≤12 years and >12 years of education, hereafter referred to as household education. A dichotomous variable, birthplace, was coded as born in Sweden (0) and born outside of Sweden (1) with information on birth country from FlexQ. Information on vitamin D supplement use (yes or no) was generated as previously described^(10)^. In short, the information on supplement use was reported in FlexQ and information on the reported use of multivitamins containing vitamin D and vitamin D supplements were utilised. A dichotomous variable (no/yes) indicating having traveled to a sunny location during the last couple of months was also generated. Residence was defined as living in an urban compared to a rural setting, which was based on the classification of the municipalities in which the participating schools were located, as described by Moraeus *et al.* ^(20)^. The dichotomous variable indicating the season for the blood collection was generated using sample date; samples collected between November and March were considered winter samples and samples between April and October were considered summer samples. Since this is a school-based study, samples from June through August are missing. A three-level variable, called geographical location, representing living in the south, middle or north of Sweden was generated based on where the participants lived. Weight was measured to the nearest 0·1 kg using SECA 862 or 899 digital weighing scales. Height was measured to the nearest 0·1 cm using SECA 213 portable stadiometers. BMI was calculated (kg/m^2^), and the International Obesity Task Force reference, taking sex and age into account was used to determine weight status^(21)^.

## Measurement of physical activity

Physical activity and sedentary time were objectively measured using tri-axial accelerometers from ActiGraph models GT3X and GT3X+ (Actigraph LLC). Study personnel distributed the accelerometers during the school visit. The participants wore the accelerometer around the waist, placed on the right hip for seven consecutive days at all times when awake, except during water-related activities. The software ActiLife Data Analysis, version 6.13.3 was used to process the accelerometer data. The monitors were set to collect data at 5-s epoch time intervals at a sample rate of 30 Hertz. Non-wear time was removed, defined as 60 min of consecutive zeros allowing for 2 min of non-zero interruptions. Participants with at least 500 min of activity registration were included in the analyses. A time filter was set between 06.00 and 22.59 for those aged 12 and 15 years and 06.00 and 23.59 for those aged 18 years. The counts from the accelerometer data were categorised into minutes spent in sedentary intensity (0–100 counts/min) and moderate to vigorous physical activity (MVPA) (≥ 2296 counts/min)^(22,23)^. The weekly average of ≥ 60 min of MVPA per day was used to categorise the participants into reaching or not reaching the physical activity recommendation. Data from the accelerometer measurements were available from 2419 participants in the total sample and from 839 participants (76 %) with measured total plasma 25(OH)D concentration. There were more valid readings from girls (*n* 485). For the present analysis, the variables counts per minute, sedentary time and MVPA were utilised.

## Statistical analysis

All analyses were performed using Stata Statistical Software Release 14. StataCorp LP. A *P*-value <0·05 was considered significant. Shapiro–Wilk’s test was used to investigate the normality of the data. The relation between plasma 25(OH)D concentration and estimated vitamin D intake were investigated with partial correlation analysis, adjusting for school year and sampling season. Food consumption and nutrient data were transformed from current intake to habitual intake using the statistical method, Multiple Source Method^(24,25)^. The transformation was stratified by school year since energy intake differed between the school years. In the Multiple Source Method model, all participants were assumed to be consumers except for fish, for which information on the consumption from FlexQ was incorporated^(20)^. Differences in plasma 25(OH)D concentration were investigated with *t* test or ANOVA according to the following categorical variables: school year, sex, household education, International Obesity Task Force weight status, residence in a rural compared to urban setting, birth place, MVPA, traveled to a sunny location during the last 2 months, sampling season, geographical location, taking a vitamin D supplement and misreporting status. All differences were stratified by school year since participants in the three school years differ in terms of development, growth and other habits, including diet. Adjusted means and corresponding 95 % CI according to vitamin D status were computed for the following variables: foods that contributed the most to vitamin D intake (fortified dairy products (35 %), fish and shellfish (11 %), red and processed meat (6 %), poultry (6 %), eggs (3 %) and fats and oils (14 %)^(26)^), as well as estimated vitamin D intake, energy intake, counts per minute and sedentary time. The adjusted means were standardised for school year and school unit, and stratified on sex.

In posthoc analysis, we built logistic regression models to identify factors that associated with vitamin D status <30 nmol/l or <50 nmol/l. In the models, respective vitamin D status was treated as the outcome, and those bivariate variables that were identified as different according to the plasma 25(OH)D concentration in previous analyses as the exposures: birthplace, household education, season of sampling, residence, sunny vacation in the previous 2 months and vitamin D supplement use. The multivariable model adjusted for sex, school year, sedentary time, vitamin D supplement, household education, season of sampling, residence, sunny vacation in the previous 2 months and vitamin D intake per 10 MJ, excluding the exposure variable in the respective model. Sedentary time was only available for 76 % of the participants thus the logistic regression models were rerun as sensitivity analysis without taking sedentary time into account. Further, differences in usual food consumption and nutrient intakes as well as sedentary time between those born in Sweden and elsewhere were investigated with *t* test. Sample weights are available in the present survey but were not included in the analyses since it was concluded previously^(13)^ that the study sample was similar to the underlying population frame.

## Results

Characteristics of study participants divided by school year and sex are presented in Table 1. The mean age in school year 5, 8 and 11 were 11·5, 14·5 and 17·9 years, respectively. Between 15 and 28 % of the participants were overweight or obese, with the highest prevalence among the oldest boys. A majority of the participants lived in an urban area, and most participants were born in Sweden. The proportion of acceptable reporters differed between 72 and 59 % with the lowest proportion among boys in school year 8, but the mean energy intake was not underreported. Usual intakes of nutrients per day were not drastically different between the different age groups. Mean intake of added sugar (energy percent (E%)) was the highest (11·9 E%) among boys in school year 8, protein intake was the highest among boys in school year 5 (17·7 E%) and girls in school year 5 had the highest vitamin D intake per 10 MJ (7·3 µg). Girls had in general higher usual intakes per 10 MJ of vitamin C, folate and Fe, but lower usual Ca intakes per 10 MJ. Boys in school year 5 spent the longest mean time in MVPA per day (59 ± 20 min) and boys in school year 11 had the longest mean sedentary time per day (658 ± 88 min). The prevalence of vitamin D insufficiency (25(OH)D concentration < 50 nmol/l) was 32 % in boys and 48 % in girls in the youngest age group. In school year 11, 62 % of the boys and 43 % of the girls had vitamin D insufficiency. The prevalence of deficiency (25(OH)D concentration < 30 nmol/l) was 16 and 15 % in boys and girls, respectively. Only 79 individuals had a plasma 25(OH)D concentration > 75 nmol/l (data not shown).

**Table 1:** Characteristics among participants divided by school year and sex

	Year 5				Year 8				Year 11			
	Girls		Boys		Girls		Boys		Girls		Boys	
	*n*	%	*n*	%	*n*	%	*n*	%	*n*	%	*n*	%
Number of participants	166		165		232		178		222		137	
Age (years)												
Mean	11·5		11·6		14·5		14·5		17·8		17·9	
sd	0·4		0·4		0·4		0·3		0·7		0·7	
IOTF weight status, *n* (%)												
UW/NW	129	78	127	77	191	82	151	85	169	76	98	72
OW/OB	37	22	38	23	41	18	27	15	53	24	39	28
Household education > 12 years, *n* (%)	97	60	97	61	150	68	113	66	112	53	73	59
Urban residence, *n* (%)	102	61	114	69	176	76	114	64	111	50	82	60
Birth place, *n* (%) Sweden	150	90	146	88	213	92	163	92	192	86	114	83
Total plasma 25(OH)D (nmol/l)	50·4	13·9	55·5	14·9	52·6	17·4	53·6	15·8	53·3	21·7	46·5	15·9
Estimated proportion												
Vitamin D deficiency (<30 nmol/l)												
%	8·4		6·1		8·2		6·8		15·3		16·1	
95 % CI	5·1, 13·8		3·3, 10·9		5·3, 12·5		3·9, 11·5		11·1, 20·7		10·8, 23·2	
Vitamin D insufficiency (<50 nmol/l)												
%	47·6		32·1		43·1		37·6		42·8		62·0	
95 % CI	40·1, 55·2		25·4, 39·7		36·9, 49·6		30·8, 45·0		36·4, 49·4		53·6, 69·8	
Sunny holiday during last 2 months, *n* (%)	25	15	15	9	29	13	20	11	24	11	14	10
Winter sample, *n* (%)	87	52	87	52	144	62	104	58	94	42	64	47
Acceptable reporters, *n* (%)	95	66	82	66	127	65	79	59	96	65	69	72
	Mean	sd	Mean	sd	Mean	sd	Mean	sd	Mean	sd	Mean	sd
Usual intakes per day, mean (sd), *n* 1100												
Vitamin D (µg)	5·8	2·2	5·9	2·3	5·9	2·0	7·6	2·9	4·8	1·7	6·3	2·5
Vitamin D (µg per 10 MJ)	7·3	2·4	7·1	2·3	7·0	2·2	7·0	2·3	5·7	1·9	6·1	2·3
Ca (mg per 10 MJ)	1278	322	1291	318	1248	311	1312	384	1084	288	1099	336
Vitamin C (mg per 10 MJ)	94	38	81	35	95	44	79	42	90	37	83	38
Folate (µg per 10 MJ)	314	66	296	56	313	77	295	72	306	82	286	65
Fe (mg per 10 MJ)	9·6	1·5	9·5	1·5	9·7	2·0	9·3	1·9	9·6	1·8	9·3	1·6
Fat (E %)	34·4	3·6	34·8	4·0	35·5	4·1	34·6	4·1	36·6	4·4	35·7	4·5
Saturated fat (E %)	13·7	1·7	13·5	1·9	14·0	2·1	13·8	2·1	13·7	2·3	13·5	2·1
Monounsaturated fat (E %)	13·1	1·8	13·3	2·0	13·6	2·1	13·1	2·0	14·2	2·1	14·0	2·3
Polyunsaturated fat (E %)	4·5	0·7	4·6	0·8	4·8	0·9	4·5	0·9	5·1	0·9	4·8	0·9
Carbohydrate (E %)	47·2	4·2	46·0	4·9	47·4	4·8	46·1	5·3	45·7	45·3	44·2	5·8
Added sugar (E %)	10·0	3·1	9·3	3·4	11·3	3·3	10·2	3·4	11·2	3·7	10·1	3·8
Fibre (g/10 MJ)	21	4·3	21	4·4	22	6·0	20	5·2	22	6·2	19	5·0
Whole grains (gram per 10 MJ)	39	21	38	23	36	21	32	20	39	24	31	17
Protein (E%)	17·3	2·5	17·7	2·5	16·2	2·3	17·2	2·8	15·7	2·6	17·4	3·3
Energy (MJ)	7·9	1·4	8·4	1·9	8·6	1·9	10·9	2·7	8·6	2·1	10·6	2·4
Physical activity measurements, *n* 839, mean (sd) if not otherwise indicated												
Counts per minute	487	165	484	174	362	115	420	157	346	121	391	166
Moderate to vigorous PA level, minutes per day	55	16	59	20	47	16	54	5	47	18	53	23
Sedentary PA level, minutes per day	485	72	485	70	648	68	626	73	656	71	658	88
Meeting recommendation of more or equal to 60 min of MVPA per day												
*n*	45		56		38		52		28		35	
%	31		45		20		39		19		36	

IOTF, International Obesity Task Force; UW/NW, underweight/normal weight; OW/OB, overweight including obese; E%, energy percent; PA, physical activity; MVPA, moderate to vigorous physical activity.

There was overall a positive correlation, adjusted for sampling season and school year between the usual intake of vitamin D (µg/d) and total plasma 25(OH)D (nmol/l) (*r* = 0·13, *P* < 0·001) among all participants and the correlation was slightly stronger in boys (*r* = 0·18, *P* < 0·001) compared with girls (*r* = 0·10, *P* < 0·05). There was overall no difference in total plasma 25(OH) D concentration between girls (mean 52·3 (sd 18·3) nmol/l) and boys (mean 52·2 (sd 15·9) nmol/l), *P* = 0·97. Differences in 25(OH)D concentration according to different background and lifestyle characteristics and stratified on school year are presented in Table 2. The factor that was associated with the largest difference in 25(OH)D concentration was country of birth. Those participants who reported their birth country to be outside of Sweden had significantly lower plasma 25(OH)D concentrations in all three school years (*P* < 0·001), with the lowest levels among those attending school year 11. Syria, Afghanistan and Iraq were the most common birth countries outside of Sweden. The proportion of participants with a birth place outside of Sweden was between 8 % in school year 8 and 17 % among boys in school year 11 (Table 1). Other characteristics that were associated with lower plasma 25(OH)D concentration were being a girl, living in a household with a shorter educational (both school years 5 and 11) attainment, and winter sampling among the youngest participants. Being a supplement user but also residence in a rural location were, only among participants in school year 8, associated with a higher plasma 25(OH)D concentration. Altogether 127 participants indicated a trip to a sunny location during the last 2 months before the survey, and this was associated with a higher plasma 25(OH)D concentration in school year 8 and 11. The largest difference was found among participants in school year 11, 63·4 ± 3·9 compared with 49·3 ± 1·1 nmol/l.

**Table 2:** Concentration of total plasma 25(OH)D by different characteristics and school year

		School year 5				School year 8				School year 11			
		*n*	Mean nmol/l	sd	*P* for difference	*n*	Mean nmol/l	sd	*P* for difference	*n*	Mean nmol/l	sd	*P* for difference
School year		331	52·9	14·6	–	410	53·0	16·7	–	359	50·7	20	0·12
Sex	Girls	166	50·4	13·9	0·002	232	52·6	17·4	0·58	222	53·3	21·7	0·002
	Boys	165	55·5	14·9		178	53·6	15·8		137	46·5	15·9	
Household education	<12 years	126	50·6	15·1	0·03	127	52·5	15·8	0·52	150	49·0	19·1	0·03
	>12 years	194	54·3	13·9		263	53·6	16·9		185	53·8	20·2	
IOTF weight status	Underweight/normal weight	256	53·3	14·4	0·36	342	53·2	16·2	0·62	267	50·9	19·9	0·78
	Overweight/obese	75	51·6	15·5		68	52·1	19·3		92	50·2	20·4	
Obesity	Yes	9	53·0	14·6	0·60	11	53·0	16·8	0·79	23	51·1	20·2	0·18
	No	332	50·4	16·0		399	51·7	41·0		336	45·3	14·9	
Residence	Urban	216	53·1	14·4	0·76	290	52·0	17·0	0·04	193	51·4	19·0	0·48
	Rural	115	52·6	15·1		120	55·6	15·7		166	49·9	21·1	
Birth place	Sweden	296	54·6	13·5	<0·001	376	54·0	16·2	<0·001	306	54·2	18·5	<0·001
	Outside of Sweden	34	38·9	16·1		32	40·9	18·0		52	30·6	15·9	
60 min MVPA	Yes	101	53·5	14·7	0·21	90	55·5	16·4	0·13	63	54·4	19·1	0·26
	No	168	51·2	14·6		237	52·4	17·3		180	51·1	20·2	
Sunny holiday*	Yes	40	53·1	0·86	0·52	49	59·8	1·76	0·003	38	63·4	3·9	<0·001
	No	291	51·5	0·80		357	52·3	0·90		319	49·3	1·1	
Sampling season	Summer	157	54·8	14·8	0·03	162	54·9	16·1	0·07	201	51·1	21·2	0·70
	Winter	174	51·9	17·1		248	51·9	17·1		158	50·2	18·3	
Geographical location in Sweden	North	44	55·0	14·1	0·36	17	56·0	23·4	0·30	69	49·6		0·13
	Middle	122	51·6	14·7		148	54·4	16·2		113	53·8	21·3	
	South	165	53·4	14·7		245	52·0	16·5		177	49·1	19·0	
Misreporting status	Under reporters	47	49·6	15·5	0·43	36	51·1	19·7	0·24	23	50·9	20·5	0·97
	Acceptable reporters	177	52·7	14·8		206	54·5	17·0		165	52·1	19·4	
	Over reporters	45	52·1	13·2		85	51·2	15·6		55	52·0	21·6	
Vitamin D supplement user	Yes	15	51·7	12·1	0·73	30	66·5	22·3	<0·001	20	56·1	26·5	0·21
	No	316	53·0	14·7		380	52·0	15·8		339	50·4	19·5	

IOTF, International Obesity Task Force; MVPA, moderate to vigorous physical activity per day.

* Sunny holiday during the last 2 months.

When the usual consumption of foods that contributed the most to vitamin D intake, estimated vitamin D and energy intake, as well as counts per minute and sedentary time were stratified according to vitamin D status, the following were observed. (Table 3). Participants, both girls and boys, that had a plasma 25(OH)D concentration below 30 nmol/l had a higher sedentary time as well as a lower consumption of dairy products and fats and oils. Among girls, a lower intake of red and processed meats, a lower count per minute and a lower intake of vitamin D (absolute intake and per 10 MJ) was associated with a plasma 25(OH)D concentration below 30 nmol/l. In boys, a plasma 25(OH)D concentration above 50 nmol/l was associated with higher counts per minute as well as a high intake of dairy products.

**Table 3:** Adjusted means and 95 % CI of background and diet variables according to vitamin D status among girls and boys. The adjusted means takes school year and school unit into account

	Vitamin D status	Girls		Boys	
		Mean	95 % CI	Mean	95 % CI
Total plasma25 (OH) (nmol/l)	0 (<30 nmol/l)	22·3	21·4, 23·3	23	22·9, 23·7
	1	41·1	40·5, 41·6	41·5	40·7, 42·2
	2 (>50 nmol/l)	64·2	62·8, 65·5	62·1	61·0, 63·2
Counts per minute	0	343	329, 357	402	386, 418
	1	392	377, 407	400	382, 419
	2	411	399, 423	460	439, 480
Sedentary time (min/d)	0	670	655, 685	640	634, 646
	1	636	628, 643	626	618, 634
	2	618	612, 625	615	606, 624
Energy intake (MJ)	0	8·4	8·0, 8·7	9·3	9·1, 9·5
	1	8·4	8·1, 8·7	10	9·7, 10
	2	8·5	8·4, 8·7	10	9·9, 10
Vitamin D (µg)	0	4·6	4·3, 4·9	5·5	5·3, 5·7
	1	5·6	5·4, 5·9	6·4	6·1, 6·7
	2	5·9	5·6, 6·1	6·9	6·7, 7·2
Vitamin D (µg/10 MJ)	0	5·8	5·4, 6·1	6·2	6·0, 6·3
	1	6·8	6·6, 7·1	6·4	6·1, 6·7
	2	6·9	6·7, 7·2	6·9	6·7, 7·2
Dairy products (g/d)	0	201	171, 232	204	192, 216
	1	281	261, 301	368	331, 406
	2	344	322, 367	480	449, 511
Eggs (g/d)	0	6·4	4·4, 8·4	12·5	9·4, 16
	1	6·3	3·5, 9·1	11·8	9·5, 14
	2	8·7	6·9, 10·5	8·2	6·3, 10
Fats and oils (g/d)	0	5·5	4·6, 6·4	7·3	6·6, 8·0
	1	8·3	7·7, 9·0	9·8	8·8, 11
	2	8·9	8·3, 9·6	9·2	8·4, 10
Red and processed meat (g/d)	0	76	72, 81	99	93, 105
	1	87	83, 92	107	102, 112
	2	85	81, 89	115	110, 119
Poultry (g/d)	0	33	31, 36	37	33, 40
	1	30	28, 33	31	29, 34
	2	30	29, 32	34	31, 36
Fish and shellfish (g/d)	0	20	17, 23	26	25, 28
	1	23	21, 25	27	25, 29
	2	23	21, 24	25	24, 27

Vitamin D status was based on total plasma 25 (OH) concentrations and were coded as 0 < 30 nmol/l, 1 between 30 and 50 nmol/l and 2 > 50 nmol/l. Comparisons shaded in light grey were statistically different between status groups.

The logistic regression analysis revealed that the odds of having a lower vitamin D status was several fold higher for those born outside of Sweden (Table 4) and the multivariable adjusted OR for a plasma 25(OH)D concentration below 30 nmol/l was 12·6. The only other variable that was related to having a plasma 25(OH)D concentration below 50 nmol/l was sampling season. A winter sample, compared with a summer sample, gave an OR (95 % CI) of 1·32 (1·04, 1·7) in the crude model and OR = 1·53 (95 % CI: 1·13, 2·1) in the adjusted model. Having been on a sunny vacation in the last 2 months was also related to a lower odds of having plasma 25(OH)D concentration below both 50 and 30 nmol/l. All other exposures in the logistic regression models gave non-significant results. Usual food consumption and nutrient intakes, as well as sedentary time stratified according to birth country, are depicted in Table 5. Absolute intakes of vitamin D, Ca, Ca per 10 MJ and energy, as well as the consumption of dairy products (mainly milk and milk products), fats and oils, and red and processed meats were higher among participants born in Sweden compared with elsewhere. Participants born outside of Sweden reported a higher consumption of eggs and poultry. The sedentary time did not differ between the two groups.

**Table 4:** The OR of being classified as having a plasma 25(OH)D concentration below 30 and 50 nmol, according to background characteristics among the participants in Riksmaten adolescents 2016–2017

	Crude			Fully adjusted			Fully adjusted, excluding sedentary time		
	OR	95 % CI	*P*-value	OR	95 % CI	*P*-value	OR	95 % CI	*P*-value
Vitamin D supplement use (yes/no)									
<50 nmol/l	1·34	0·80, 2·3	0·27	1·41	0·75, 2·7	0·29	1·3	0·76, 2·4	0·32
<30 nmol/l	2·40	0·74, 7·8	0·14	2·93	0·6, 13·9	0·17	2·7	0·74, 9·6	0·13
Household education (<12 years/12 years)									
<50 nmol/l	0·79	0·62, 1·0	0·07	0·87	0·64, 1·2	0·40	0·83	0·63, 1·1	0·18
<30 nmol/l	0·75	0·49, 1·1	0·18	1·34	0·75, 2·4	0·32	1·08	0·66, 1·8	0·75
Residence (urban/rural)									
<50 nmol/l	0·91	0·7, 1·3	0·48	0·93	0·68, 1·3	0·64	0·86	0·65, 1·1	0·27
<30 nmol/l	1·49	1·0, 2·2	0·05	1·51	0·87, 2·6	0·15	1·4	0·87, 2·3	0·16
Sampling season (summer/winter)									
<50 nmol/l	1·32	1·04, 1·7	0·03	1·53	1·13, 2·1	0·01	1·6	1·2, 2·0	0·001
<30 nmol/l	0·83	0·56, 1·2	0·37	1·17	0·67, 2·1	0·59	1·3	0·83, 2·2	0·23
Birthplace (Sweden/elsewhere)									
<50 nmol/l	6·1	3·8, 9·7	<0·001	5·7	3·2, 10·1	<0·001	5·7	3·2, 10·1	<0·001
<30 nmol/l	12·9	8·3, 20·3	<0·001	12·6	6·75, 23·5	<0·001	13·1	7·7, 22·2	<0·001
Sunny vacation (no/yes)									
<50 nmol/l	0·61	0·4, 0·9	0·02	0·69	0·42, 1·1	0·13	0·59	0·39, 0·90	0·02
<30 nmol/l	0·49	0·2, 1·1	0·08	0·14	0·03, 0·63	0·01	0·27	0·1, 0·73	0·01

The odds ratios were calculated in logistic regression analysis. The adjusted model included, sex, school year, sedentary time, vitamin D supplement use, household education, residence, vitamin D intake per 10 MJ and misreporting status.

**Table 5:** Sedentary time and usual intakes of food groups and nutrients among participants born outside of Sweden compared to those born in Sweden

	Born outside of Sweden			Born in Sweden			*P* for difference
	*n*	Mean	95 % CI	*n*	Mean	95 % CI	
Vitamin D (µg)	118	5·5	5·0, 6·0	978	6·0	5·9, 6·2	0·04
Vitamin D (µg per 10 MJ)	118	6·6	6·1, 7·0	978	6·7	6·6, 6·8	0·43
Ca (mg)	118	983	905, 1060	978	1121	1094, 1148	<0·001
Ca (mg per 10 MJ)	118	1148	1087, 1209	978	1227	1205, 1248	0·02
Vitamin C	118	74	68, 81	978	78	76, 80	0·30
Vitamin C (mg per 10 MJ)	118	88	81, 95	978	88	85, 90	0·88
Folate (µg)	118	264	247, 280	978	273	267, 278	0·29
Folate (µg per 10 MJ)	118	310	298, 323	978	302	298, 307	0·24
Fe (mg)	118	8·2	7·7, 8·6	978	8·6	8·4, 8·7	0·09
Fe (mg per 10 MJ)	118	9·7	9·4, 10	978	9·5	9·4, 9·6	0·31
Sedentary time, min/d	83	637	617, 657	753	626	620, 631	0·21
Energy intake (MJ)	118	8·5	8·1, 8·9	978	9·1	9·0, 9·3	0·006
Usual intakes per day, gram per 10 MJ							
Dairy products	118	314	269, 358	978	388	371, 405	0·004
Eggs	118	17	12, 22	978	8	7, 9	<0·001
Fats and oils	118	7·5	6·4, 8·7	978	9·7	9·3, 10·2	0·001
Red and processed meat	118	97	88, 106	978	108	105, 110	0·01
Poultry	118	45	40, 50	978	37	36, 38	<0·001
Fish and shellfish	118	29	25, 33	978	27	25, 28	0·17

*P* for differences was tested with *t* test.

## Discussion

The results of the present study, utilising data collected in a representative cross-sectional survey among adolescents in Sweden, showed no overall difference in plasma 25(OH)D concentration between girls and boys. However, there was large variation in the prevalence of vitamin D insufficiency (plasma 25(OH)D concentration < 50 nmol/l^(1)^) between the different age groups. In boys attending school year 5, this proportion was 32 %, while among boys attending school year 11, the corresponding figure was 62 %. The proportion of participants with deficiency (plasma 25(OH)D concentration < 30 nmol/l^(1)^) was 6 % among boys in school year 5, while 15 and 16 % among year 11 girls and boys, respectively. This is consistent with a lower vitamin D density (vitamin D intake per 10 MJ) of the diet among the participants in school year 11; however, other factors appeared to be associated with vitamin D status as well. The variable that had the greatest influence on plasma 25(OH)D concentration in the present study was country of birth, a result that was independent of sex, school year, sedentary time, vitamin D supplement use, household education, residence, sampling season, a sun trip in the previous 2 months and vitamin D intake. Syria, Afghanistan and Iraq were the most common countries of origin for those born outside of Sweden. The proportion of participants born outside of Sweden was the highest in school year 11 compared with the other school years, possibly impacting the proportion of vitamin D deficient participants. The odds of a plasma 25(OH)D concentration below 30 nmol/l was above 10-fold in those born outside of Sweden, compared with those born in Sweden. We could also see that both girls and boys that had a plasma 25(OH)D concentration below 30 nmol/l had higher sedentary time as well as a lower consumption of dairy products and fats and oils, both food products with mandatory vitamin D fortification. A higher vitamin D status among the participants was found to be associated with a vacation to a sunny location in the previous 2 months.

It is known that people with a darker skin colour requires longer sun exposure time to achieve the same vitamin D concentration as people with a more fair complexion and that vitamin D deficiency is common in the Middle East^(3)^. Observations from the USA have shown that the prevalence of vitamin D deficiency and insufficiency is higher in population groups with darker skin colour^(27)^. A previous study conducted at two study sites in Sweden (north and south) among younger children (aged 5–7 years) found that the main risk factor for insufficient 25(OH)D status (<50 nmol/l) was a darker skin colour, an observation that was independent of dietary vitamin D intake^(28)^ and consistent with the observation in the present study. The present results further corroborate the results in the study conducted among 10–12-year-old school children in Sweden, that reported the lowest 25(OH)D status among those born outside of Europe^(8)^.

Studies from different Western countries have reported on differing total 25(OH)D levels in children and adolescents of diverse backgrounds. A large cohort study from the UK using data from 700 000 children aged 0–17 years, primarily studying incidence rate of vitamin D deficiency (<25 nmol/l) from primary care records, reported that vitamin D deficiency was more common among children of non-white ethnicity, in older children, and lower socio-economic position^(29)^, similar to findings of the present study. In another study (348 healthy women aged 16–25 years in Australia), the mean serum 25(OH)D concentration was 68 (sd 27) nmol/l. 26 % of the participants were classified as vitamin D deficient (<50 nmol/l in Australia), which is a lower prevalence than in the present study among the oldest girls, but the study also reported a marked difference in levels of 25(OH)D among women born in Australia compared with elsewhere. Lower levels were also related to lower educational and physical activity levels^(30)^. This observation is consistent with the results from the national Australian Health Survey among adolescents and young adults (aged 12–24 years)^(31)^. Major predictors of vitamin D deficiency were being born outside of Australia, season, overweight and low physical activity^(31)^. Another study based on national data from Canada among children aged 6–18 years also reported on lower 25(OH)D concentration among children of non-white ethnicity. These children, compared to children with white ethnicity, consumed fish and eggs more frequently while red meat less often^(32)^. This is partly similar to the findings in the present study. However, in the present study, the difference in fish intake was not statistically different in those born in Sweden compared to elsewhere. Nevertheless children born in Sweden had a higher intake of dairy products (mainly milk and milk products), the food group that contributed the most to the vitamin D intake among the present adolescents, but a lower intake of fats and oils also contributing to the vitamin D intake^(26)^. However, the difference in food consumption between those born in Sweden compared to elsewhere only partly explain the differences in 25(OH)D concentration. The absolute intake of vitamin D was lower in the participants born outside of Sweden, but in relative terms (per 10 MJ) the vitamin D intake did not differ between the groups. This means that those born outside of Sweden reported a lower energy intake, which could indicate underreporting in this group, but could also imply a lower energy requirement since this group also reported a longer time spent in sedentary intensity (however not statistically different from those born in Sweden). In the Canadian study, the 25(OH)D concentration was measured in two population samples in 2007–2009 and in 2012–2013. Between the two investigations, the 25(OH)D concentrations declined despite similar characteristics among the participants and an increased vitamin D recommendation in 2010. It was concluded that public health strategies focussing on dietary intake may not be an effective sole strategy to achieve vitamin D sufficiency among children^(32)^. This may be true in Sweden also, since there are many factors that contribute to vitamin D status. Since the Riksmaten adolescents 2016–2017 survey was conducted, the new policy on mandatory vitamin D fortification was implemented and at the time of the study, some companies had already started with the higher fortification level. The level of fortification for consumption milk (≤ 1·5 g fat content) and margarine and fat blends was almost doubled in the new legislation^(33)^. Further, it became mandatory for food producers to fortify several other categories of milk products and fat blends. It will be important to compare the present results with the current vitamin D status among adolescents living in Sweden in a follow-up study. It is important to investigate if the fortification policy is as effective in all population groups, since it is possible that different population groups choose different types of foods. In Finland, for example, the plasma 25(OH)D concentrations in the population have increased since the introduction of a voluntary fortification policy^(34)^.

In a previous study conducted among adults in Sweden, the proportion of participants with a 25(OH)D concentrations < 30 nmol/l was only 3 %, and there was no difference between men and women^(8)^. It appears that the higher prevalence of deficiency in school year 11 observed in the present study, may become lower in adulthood, since it is known that puberty is driven by inflammatory stimuli, which may lead to lower vitamin D status^(3)^. However, it should be kept in mind that the study among adults was mainly based on a homogenous group of adults mainly born in Sweden^(8)^.

The strengths of the present study include that the study sample was representative and included a large number of children and adolescents from all parts of Sweden. The study had collected specific information on food consumption and vitamin D status was measured with a method that is accredited by the Vitamin D External Quality Assessment Scheme, which also are strengths. Another strength is the use of an objective method to measure physical activity and sedentary time. The cross-sectional character of the study impedes any causal inferences to be done. However, for the objective of the present study, a cross-sectional study suitable. Dietary assessment is inherently prone to misreporting, however, misreporting status was not associated with differences in vitamin D status in the present study. However, there is still the possibility of misclassification of participants because of under- or overreporting. The proportion reaching the recommendation of physical activity was higher in the present sub-population compared with the entire study sample^(35)^, which may indicate that some selection bias may have occurred. Further, the group born outside of Sweden was rather small, which deterred us from doing more extensive analyses. The study did not collect information about neither skin type nor on sun exposure data, which are weaknesses. Blood samples were not collected during the summer months because of the study’s school-based design, however this is a weakness of the study. In the food composition database, vitamin D content of foods is reflective of the content of D2 and D3. There is, however, lack of information on the content of 25(OH)D in foods that contains it (meat and egg).

In conclusion, the results of the present representative survey of adolescents in Sweden, showed that the prevalence of vitamin D insufficiency and deficiency varied between the three school years. Participants born outside Sweden, here mainly in the Middle East, had the lowest vitamin D levels. The results of the present study will form a baseline for future follow-up studies to evaluate the effects of the increased and mandatory vitamin D fortification of foods in Sweden.
